# PReS-FINAL-2213: Validation of inadequate drug response and definition of colchicum resistance in FMF

**DOI:** 10.1186/1546-0096-11-S2-P203

**Published:** 2013-12-05

**Authors:** E Demirkaya, C Acikel, G Basbozkurt, A Gul, O Kasapcopur, O Aydog, H Erdem, A Duzova, B Kisacik, T Kasifoglu, E Erken, M Tunca, M Sayarlioglu, S Yuksel, F Yildiz, O Donmez, A Berdeli, S Senel, NA Ayaz, A Polat, B Sozer, Y Tabel, S Akar, AM Onat, O Ozkaya, S Emre, N Akinca, G Ozcelik, S Yavuz, S Yesilkaya, F Gok, HM Poyrazoglu, H Direskeneli, S Bakkaloglu, S Erten, A Tufan, B Goker, S Kavukcu, N Cakar, M Saldir, A Delibas, B Makay, A Kısaarslan, SE Unsal, H Ozdogan, R Topaloglu, S Ozen

**Affiliations:** 1 FMF Arthritis Vasculitis and Orphan disease Research in Pediatric Rheumatology (FAVOR), Ankara, Turkey

## Introduction

Colchicine is effective in controlling the attacks and preventing the development of amyloidosis. Moreover many new generation drugs may be used in FMF treatment. About 5-10% of the patients do not respond to colchicine and inadequate response to other drugs is not as clear as the unresponsiveness of colchicine.

## Objectives

In this study it was aimed to determine the set of criteria for the diagnosis of inadequate drug response in FMF.

## Methods

This study was based on a Delphi survey. Open-ended questions were sent to 70 experts on FMF. In the first Delphi round, clinical and laboratory findings indicating colchicine resistance and the protocol, which would define resistance to treatment and exclusion criteria, were asked. Based on the results of the first Delphi, a second Delphi form, which included 5 evaluation questions, was developed. In this latter form the questions to be used in order to define complete response, partial response and non-response were tried to be determined. As a last step, a consensus meeting held on by attendance of 12 (6 of pediatric rheumatologist and 6 of adult rheumatologist) experts. The experts evaluated all candidate variables; discuss weighting methods and exact expressions of questions.

Before consensus meeting each expert sent at least 6 selected patients to be used in validation. Physicians gave severity, activity, and drug response scores for all cases. After determination of criteria set, inadequate drug response scores were calculated in this data set. Binary logistic regulation analyses were performed to evaluate value of criterion, and ROC curves were drawn to present validity.

## Results

For the validation study we collected 297 patients from 23 centers in Turkey. According to consensus meeting; number of attacks, duration of attacks, VAS score of physicians, VAS score of patients, persistence of arthritis, arthralgia, and myalgia, and high CRP levels were selected the best predictors for inadequate response to treatment in patients with FMF. Persistence of arthritis was eliminated in logistic regression analyses. Area under the curve calculated as 0.829 for full model and 0.816 for the model that persistence of arthritis eliminated from criteria set.

## Conclusion

Assessing the colchicine resistance via concrete and agreed scale will provide a reliable data. The criteria set considerate as valid for evaluation of inadequate drug response. There is no statistically difference between 6 items and 7 items criteria sets. On the other hand recovery of arthritis maybe more valuable among adult patients. A prospective validation study will be conducted with daily outpatient cases.

## Disclosure of interest

None declared.

